# Cycloid psychosis - from the past to the future: based on a case report

**DOI:** 10.1192/j.eurpsy.2022.2055

**Published:** 2022-09-01

**Authors:** S. Pereira, A. Almeida, J. Pais

**Affiliations:** Centro Hospitalar do Tâmega e Sousa, Psiquiatria E Saúde Mental, Gilhufe - Penafiel, Portugal

**Keywords:** cycloid psychosis, brief psychotic disorder, Acute and transient psychotic disorder, atypical psychosis

## Abstract

**Introduction:**

The concept of cycloid psychosis has a long tradition in European psychiatry since it was introduced by Kleist in 1926. Nevertheless, this concept is not included explicitly in modern classifications, leading to a controversial discussion about its utility in current psychiatry.

**Objectives:**

Starting from a case study, we intend to review the evolution of cycloid psychosis concept and analyze its role in modern psychiatry.

**Methods:**

Non-systematic review of the literature and report of a case study.

**Results:**

Following Kleist’s work, Leonhard described the three overlapping subtypes, and later Perris developed the first operational diagnostic criteria. Since then, this entity has shown a high diagnostic stability, validity and a good predictive diagnostic and prognostic value. We report a case of a 30-year-old woman, previous heathy, without regular medication, living with her parents and 5-year-old son, until she emigrated alone to Switzerland. After 10 days abroad, she was sent back to Portugal, and after organic disease and drug misuse exclusion, she was admitted in our inward with a clinical picture of perplexity, anxiety, thinking and behavioral disturbance with persecutory and poisoning delusions, auditory hallucinations, and total insomnia. Following rapid and full recovery, she was discharged 14 days later while being medicated with Paliperidone 3 mg/day and Lorazepam 4 mg/day, which was abandoned by her 2 months later, without relapse of the symptoms.

**Conclusions:**

The current lack of a satisfactory system for categorizing acute, and remittent psychoses seems to be reason enough to remain awareness of this unique diagnostic entity, which is worthy of further investigation.

**Disclosure:**

No significant relationships.

